# Tacit knowledge as the unifying factor in evidence based medicine and clinical judgement

**DOI:** 10.1186/1747-5341-1-2

**Published:** 2006-03-17

**Authors:** Tim Thornton

**Affiliations:** 1 Professor of Philosophy and Mental Health, Centre for Ethnicity & Health, University of Central Lancashire, Preston, UK

## Abstract

The paper outlines the role that tacit knowledge plays in what might seem to be an area of knowledge that can be made fully explicit or codified and which forms a central element of Evidence Based Medicine. Appeal to the role the role of tacit knowledge in science provides a way to unify the tripartite definition of Evidence Based Medicine given by Sackett et al: the integration of best research evidence with clinical expertise and patient values. Each of these three elements, crucially including research evidence, rests on an ineliminable and irreducible notion of uncodified good judgement.

The paper focuses on research evidence, drawing first on the work of Kuhn to suggest that tacit knowledge contributes, as a matter of fact, to puzzle solving within what he calls normal science. A stronger argument that it *must *play a role in research is first motivated by looking to Collins' first hand account of replication in applied physics and then broader considerations of replication in justifying knowledge claims in scientific research. Finally, consideration of an argument from Wittgenstein shows that whatever explicit guidelines can be drawn up to guide judgement the specification of what counts as correctly following them has to remain implicit.

Overall, the paper sets out arguments for the claim that even though explicit guidelines and codifications can play a practical role in informing clinical practice, they rest on a body of tacit or implicit skill that is in principle ineliminable. It forms the bedrock of good judgement and unites the integration of research, expertise and values.

## Introduction

In their book, *Evidence-based Medicine: How to practice and teach EBM*, David Sackett, Sharon Straus, Scott Richardson, William Rosenberg, and Brian Haynes define it as follows. 'Evidence based medicine is the integration of best research evidence with clinical expertise and patient values.' [1]

This is a surprising definition. Normally, the focus of EBM is on the first element of that tripartite division: best research evidence. But Sackett et al widen their definition to include two further aspects: expertise and values. They give a further brief preliminary sketch of each as follows.

By *best research evidence *we mean clinically relevant research... New evidence from clinical research and treatments both invalidates previously accepted diagnostic tests and treatments and replaces them with new ones that are more powerful, more accurate, more efficacious and safer.

By *clinical expertise *we mean the ability to use our clinical skills and past experience to rapidly identify each patient's unique health state and diagnosis, their individual risks and benefits of potential interventions, and their personal values and expectations.

By *patient values *we mean the unique preferences, concerns and expectations each patient brings to a clinical encounter and which must be integrated into clinical decisions if they are to serve the patient. [1]

Two preliminary points are worth making about this substantial overall definition. Firstly, it looks to be a definition of what would normally be taken to be, not EBM as such, but something that should be based on it: best clinical practice, perhaps, or clinical judgement.

Secondly, it can seem that these three elements do not form a unifiable whole, and thus the proper subject for an attempt at unified definition. Of course, to take the first element, being research evidence-based does not preclude a judgement being based on other things as well. An evidence-base and a values-base can go hand in hand in informing patient centred clinical judgement. But to either an unschooled eye, or to an eye overly schooled in positivistic models of scientific method, the three elements may look disparate for a specific reason.

One of the thrusts of EBM is to promote the use of good quality reliable evidence through hierarchies which put meta-analysis of randomised controlled trials at the top, descriptive studies lower down and respected authority (radically) at the bottom. This is an attempt to codify or apply explicit rules to judgements about evidence. By contrast, clinical expertise is not codifiable. It depends instead on skilled judgement drawing on personal experience.

Judgements about patient values may or may not seem to be codifiable depending on the view one takes of their nature. If one assumes both that the relevant values are exhausted by moral or ethical values and also that such values can be codified in principles (one adopts a 'principlist' approach) then one may think of the third aspect in just the same algorithmic way as a positivist takes the first (and thus unlike the second). Skill in following medical ethical values, on this view, is an algorithmic skill in deduction from general principles.

A more sophisticated view, however, recognises that ethical values are just one sub-set of the values that need to be taken into account in clinical judgement. Vales Based Practice, on this alternative view, should take into account the preferences, likes and dislikes of stakeholders in a judgement, aiming to balance opposing claims rather than assuming that an ideal solution exists [2]. If so, it looks more like the second aspect of EBM, clinical expertise, and unlike the first, research evidence.

But even on the mistakenly narrow assumption that only ethical values are important, it is arguable that any ethical principles that do hold only hold – in accord with Aristotle's views -for the most part. It is not possible to draw up a list of principles that are both contentful – ie can actually guide medical practice – and are mutually consistent. But if this is not possible then ethical judgements are not simply rule or principle driven. Some other factor must be in play in, eg, selecting which principle is most important in each different context which is not codified in the principles themselves [3].

It is worth considering values a little further because this will reveal an assumption that is relevant to broader consideration of tacit knowledge. One motivation for assuming that ethical values can be codified in a set of principles is, according to the philosopher John McDowell, an assumption or prejudice about the nature of rationality in general [4,5]. He suggests that Aristotle's otherwise obvious truth about the uncodifibaility of moral judgement is missed because of this. A comparison with a paradigmatically *codified *judgement should help make this clear. Consider a simple mathematical series such as counting up in two's. McDowell suggests that we tend to think of competence in continuing such a series as resulting from a psychological mechanism, an internal state, which reliably delivers the right result at each point. Because of this we expect codifications elsewhere, as well. But in fact it is a mistake to think that a psychological mechanism could explain why just the *right *numbers – out of the multitude of wrong numbers – are given for an infinite series.

The natural but mistaken picture can also be augmented, especially in mathematical cases, with the idea that the mechanism tracks or follows objective, perhaps supernatural, rails which are already out there, independently of us. Our continuing a series is, to use another metaphor, merely going over, in bolder pencil, moves already somehow made. This serves as a model of what objectivity amounts to. In medical, rather than mathematical, cases the reality is a little more straight-forward. Rather than tracking supernatural mathematical facts, objective medical judgements aim at *validity*: tracking real distinctions in our physiological nature or the nature of diseases.

With this assumption in place, it seems that the making of any genuinely objective judgement could be codified. Schematically, judgements involve applying a concept to a subject. Thus it seems that they ought to be codifiable by giving a principle or formula 'that specifies the conditions under which the concept, in the use of it that one has mastered, is correctly applied' [6]. This is a mistaken view which leads in turn to the assumption that all genuinely cognitive judgements must be governed by an explicit rule.

Now it is only this misconception of the deductive paradigm that leads us to suppose that the operations of any specific conception of rationality in a particular area – any specific conception of what counts as doing the same thing – must be deductively explicable; that is, that there must be a formulable universal principle... [6]

In other words, the view one takes of ethical judgements depends on one's view of their objectivity against a background assumption that, where there is objectivity, a codification or explicit rule for the corresponding judgement is possible. If one takes value judgements to be codifiable in this way, alongside research or evidence-based judgements, only clinical expertise, of Sackett's tripartite definition, resists explicit codification, almost by its definition. But one common interpretation of EBM is precisely to discount the contribution of expertise for that reason. What cannot be codified cannot be a proper exercise of rationality, is not an objective matter. (I will return to Wittgenstein's and McDowell's diagnosis of the mistake at the end of this paper.)

Whatever the view of value judgements, the evidence base of medicine is often taken to involve a particularly robust kind of rational judgement: judgement that can be codified in rules or guidelines. This reflects the widespread view that research is based on a scientific method that is itself explicit or codifiable. Once this assumption is in place, any aspect of clinical practice that cannot be so codified can seem arbitrary or subjective in comparison. This in turn suggests that the three aspects that make up Sackett's complex view of EBM cannot be unified. They differ fundamentally.

But this view of the immiscibility of the elements can be resisted, not by attempting to assimilate expertise to the positivistic model of research evidence and the principlist model of ethical judgements, but the other way round. In this paper, I will outline a principled reason for this approach by outlining a role for tacit knowledge in scientific practice including its role in research. This will undermine the view of evidence-based judgement as algorithmic and thus help undermine the comparison that that idea motivates. Without that view, clinical practice can be seen as unified through the idea that all three elements of Sackett's composite rest on uncodifiable judgement. Practical rationality rests on a kind of cognitive skill: *judgement *in the sense 'judgement' has in the phrase 'having good judgement'.

The discussion will draw on the history of science, the social study of scientific knowledge and pure philosophy. In so doing this example nicely illustrates the insight the humanities in general, and philosophy in particular, can shed on medicine. (The important traffic in the opposite direction will not be discussed here.)

## Kuhn's account of tacit knowledge

Although the term was first popularised by Michael Polanyi, an influential first clue to the role of tacit knowledge in EBM can be found in Thomas Kuhn's *Structure of Scientific Revolutions *[7,8]. On his familiar account, the practice of science as a whole comprises lengthy periods of 'normal science' punctuated by occasional, briefer periods of revolutionary change. Most scientists, for most of the time, are thus engaged in the activities that make up normal science.

Kuhn suggests that the main activity of normal science is 'puzzle solving' which he compares to crossword and jigsaw puzzles. Like those everyday cases, Kuhnian puzzles are assumed to be soluble and the nature of the solutions sought is highly circumscribed, in this case by the dominant theoretical background. Solving them is thus not a matter of great surprise but rather serves as a test of the theoretical or experimental prowess of the scientist in question. It also serves to extend and make more explicit the overall background set of theories and high level metaphysical assumptions which, inter alia, Kuhn labels 'paradigms'. Puzzles which mattered to previous paradigms, by contrast, may be rejected as the product of bad science or bad metaphysics or they may simply be ignored until a subsequent revolutionary change makes them into important puzzles again.

Kuhn goes on to suggest that puzzle solving highlights the role of tacit knowledge in theoretical science. One of the central skills which is acquired by puzzle solving is learning to recognise how to apply the background theories to new cases, what assumptions or approximations count as reasonable, what would constitute a satisfactory solution, and so forth. In other words, possession of explicit knowledge of a regimented theory is insufficient to be counted as a competent scientist. One must also have the 'know-how' required to *apply *high level theories to particular cases. A key element of this is to recognise that apparently different puzzles can in fact be treated in the same or analogous ways.

That is only one of the ways in which scientific research work is guided by tacit knowledge. Kuhn also suggests that scientific work is guided by a set of underlying assumptions or commitments in four ways:

• At a practical level, research is guided by commitments to particular kinds of instrument, experiment or tests.

• Laws and theories...

• ... and higher level meta-theoretical or metaphysical assumptions determine what is taken to be the subject of science, what sort of thing there is – atoms in a plenum or fields of force – and thus the sort of account to be developed.

• Finally, the values which are constitutive of being a scientist: weight placed on rationality, coherence, quantification, observation and measurement.

It might seem that these commitments are imposed upon scientists working within a particular tradition in the form of *explicit *rules or codes. But Kuhn argues that they are, in fact, implicit or tacit. He provides two main arguments for this claim. The first is empirical. Historical inquiry has simply failed to discover evidence of sufficient numbers of explicit rules to explain the coherence of scientific traditions. Therefore the rules must be *implicit*. (Note, by the way, that Kuhn himself reserves the word 'rule' for explicit rules.)

Secondly, Kuhn suggests *why *it might be that the rules are implicit. Much scientific training, from its beginnings in school-work to PhD level and beyond, is by example and application. Terms are introduced together with the theoretical context in which they have their life. Theories are introduced alongside applications in the solution of problems or puzzles. Most of a scientific education comprises learning how to apply theories to problems. If these 'finger exercises' are successful, a trainee scientist learns to see similarities between cases which permit the application of familiar puzzle solving techniques. But this does not require that he or she has abstracted an explicit rule about what it is that makes cases similar *except *that the same sort of solution can be applied.

Kuhn's account of science as a whole echoes the practical aspect of medical training. What the account adds, however, is an emphasis on the role of tacit knowledge not only in practical contexts – the application of medical theory on wards or general practice surgeries from the recognition of symptoms to the formulation of management plans – but also in furthering the research findings of scientific paradigms.

Although this account of the role of tacit knowledge in science is suggestive, it is limited in two related respects. Firstly, the most it seems to offer is a claim to the effect that, as a matter of fact or as science is presently practiced, tacit knowledge is involved. This contrasts with a stronger claim that tacit knowledge *must *be involved. Thus it might be thought that this is reason itself to regulate or codify practice through, eg, the sort of guidelines that make up EBM. But, although such guidelines can be useful, this is only in a context of practical know-how. Or so I shall argue.

Secondly, although Kuhn suggests that 'finger exercises' are important in the account of tacit knowledge, he does not provide a deep account of why this rules out explicit knowledge. Could the knowledge that, as a matter of fact, is transmitted through finger exercises be fully codified, in principle, in explicit guidelines? To remedy both these lacks I will turn in the next section first to the example of cookery and thence to another piece of empirical analysis, this time by a sociologist of knowledge, Harry Collins.

## Tacit knowledge in cookery and engineering science

In his book *Changing Order: Replication and Induction in Scientific Practice*, Harry Collins charts the role of tacit knowledge in both scientific and extra-scientific practice (eg parapsychology). As I will describe below, the idea of replication plays a central role in underpinning research evidence. Replication is a practical response to the principled problem of how one can learn from experience through induction and thus lies at the heart of EBM. But the role of tacit knowledge in that broader context can be seen more clearly by looking to a more concrete case of replication that Collins also discusses: replicating a new kind of laser. That discussion, however, can itself be clarified by starting this section with an everyday case: basic cookery.

By basic cookery I mean the kind of culinary skills acquired through both formal home economics teaching and informal apprenticeship in the home. It is the level of cookery involved in making cakes, baking bread, roasting or casseroling meat, making pancakes, scrambling eggs etc. Basic cookery is a theory-informed practical skill with normative standards. It has normative standards in the sense that it is possible to get it wrong. One can *fail *to cause a cake to rise, fail to roast chicken such that it is cooked on the inside without burning the outside, fail to mix batter of the right consistency that resultant pancakes have sufficient structural integrity. Even given the vagaries of taste, there are still culinary standards to aim for. (The additional skills of great chefs, by contrast, do not seem to be subject to such clear cut norms. This may be because such chefs are as much responsible for changing standards of taste as answering to them.)

As well as answering to normative standards, basic cookery involves both a body of practical skill and of explicit theory. The theory includes, eg, relative proportions of ingredients for the mixing of batter; cooking times, for a fixed temperature, as a function of weight of poultry; the correct appearance of molten butter for scrambling eggs etc. At their most practical, skills include how to whisk egg whites, to toss pancakes, assess whether a cake is cooked on the inside by piercing it with a skewer etc. What then is the relation between these different aspects of basic cookery? Does cookery depend on tacit knowledge and, if so, must it?

Most people have had the experience of attempting to follow a recipe, attending to each stage of the process, and yet failing to obtain the desired and predicted result, failing the relevant normative standard. Why is this? One possibility is a breakdown of the practical skills involved. Perhaps the cook lacks, eg, the physical strength or dexterity to whisk the mixture at sufficient speed. In most cases, however, especially given the mechanisation of even domestic cookery, this is not the cause of the problem. But on the – fallible – assumption that none of the explicit instructions has been violated what other explanation is there?

The most obvious explanation is that the recipe is incomplete. Perhaps a key step has been omitted even if it is one that would not need to be explicitly stated for a skilled cook (which perhaps explains its omission by its skilled author). Such a cook can 'fill in' the missing step unthinkingly whereas the novice comes unstuck. Tacit knowledge fits this picture as an abbreviation of explicit knowledge. But would it be possible to make all such knowledge explicit? In the UK, one particular cook, Delia Smith, is famous for her attempt to make culinary skill as explicit as possible in both books and television programmes. According to her *How to Cook*, there are, for example, nine stages in making a piece of toast (merely the seventh of which is to eat the toast as soon as possible) [9]. But, aside from the merely practical skills, could this approach remove all need of tacit knowledge? Could all knowledge be explicit?

Harry Collins, a sociologist of knowledge, addresses this question in his discussion of a case drawn from applied physics, or theoretical engineering. The example concerns the practical difficulties of scientific replication. In this case, 'replication' is quite literal. Collins describes an attempt to build a working laser which, although of a new design (a Transversely Excited Atmospheric pressure CO_2_, or TEA, laser), had already been successfully tested through the construction of working versions in other laboratories. The challenge is to build another one. In one case, a scientist who has already built one working model aims to replicate it so as to have two working models. Despite this limited problem – a clear case of Kuhnian 'normal science' – and despite the availability of explicit instructions, Collins discovered a surprising difficulty.

[N]o scientist succeeded in building a laser by using only information found in published or other written sources. Thus every scientist who managed to copy the laser obtained a crucial component of the requisite knowledge from personal contact and discussion. A second point is that no scientist succeeded in building a TEA-laser where the informant was a 'middle man' who had not built a device himself. The third point is that even where the informant had built a successful device, and where information flowed freely as far as could be seen, the learner would be unlikely to succeed without some extended period of contact with the informant and, in some cases, would not succeed at all...

In sum, the flow of knowledge was such that, first, it travelled only where there was personal contact with an accomplished practitioner; second, its passage was invisible so that scientists did not know whether they had the relevant expertise to build a laser until they tried it; and, third, it was so capricious that similar relationships between teacher and learner might or might not result in the transfer of knowledge. These characteristics of the flow of knowledge make sense if a crucial component in laser building ability is 'tacit knowledge'. [10]

Why might this be so? Aside from the empirical claim that this was simply what he found, Collins' detailed first hand account of one scientist's (Dr Bob Harrison's) progress contains some general lessons.

The problem scientists confront in such cases is this. Assembling a complex arrangement of component parts, even given the constraint of accord with physical theory, does not usually at first produce a working machine. If the whole machine does not work this must be because at least one of the components does not work or is connected wrongly into the whole. But in general, the only direct test of whether a component is itself working properly is its contribution to a working overall assembly, which is in turn impossible until all the components are working. Likewise, the test that any is connected properly is, ultimately, that all are correctly connected in the working machine. Collins refers to this problem as the 'Experimenters' Regress'. In the area of science that Collins discusses which is a mixture of mature and developing science (many components of the lasers are 'off the shelf', others have to be specifically made by the scientists), building instruments to test laser components would be possible. But such instruments would themselves be complex devices, mimicking or replicating the environment of the component in the laser.

Given the Experimenters' Regress, there is no simple procedure for 'debugging' the apparatus. It might be thought that the solution to the problem of debugging lies in avoiding it by carefully following published accounts. But Collins gives reason to think that these cannot be sufficient. Published explicit guidelines underdetermine the range of judgements that need to be made. In the case of replicating a working laser, there is what looks to be the most explicit guidance possible. Rather than following written instructions – which stand in for, by symbolically representing, a working laser – there is, instead, a real laser to copy. But to copy even this involves judgements of relevant similarity and dissimilarity between the original and the copy.

Throughout, Bob Harrison [the scientist] and I had been discussing similarities and differences between the old and new laser. I had noticed the different thickness of the wires and had suggested that this might be significant. One of the graduate students had agreed that the thinner wires would have a significantly reduced surface area which might prevent proper pre-ionization. Yet Harrison had failed to see this as a significant difference; as it turned out his *not seeing *the difference was the proper way to see things. They were in fact 'just wires'... There were other differences I noticed and that Harrison ignored, quite rightly as it turned out... Without knowing how to ignore all these things we might have spent months checking them out... None of the things that Harrison had learned to ignore would be obviously significant, or insignificant, on a circuit diagram or in a technical article. The range of things to be ignored is, of course, indefinitely long. On the other hand, in developing his laser building skill Harrison had also learned to see significance where previously he had noticed nothing. [11]

In a practical example of replication like this, the number of differences between the original and the copy is likely to be high. But Collins indicates a further, principled, source of complexity: the number of factors that can safely be ignored or are not critical. In the case of copying a working laser, this list is not written down anywhere. The rule is simply to copy the original. But if it were written down it would, in principle, have to include an infinite number of factors. Thus explicit knowledge of it looks to be impossible. Any statement of Harrison's knowledge – once he has acquired laser-building skill – of such factors, on which he does not waste time, will be inadequate.

In the case at hand, however, it might seem that such a list is unnecessary. If replication of a laser is a matter of making an exact copy, then there is no need for that further specification. But this raises a more general question of the role of replication in science which will be significant for assessing the role of tacit knowledge in research evidence. In general, in science what matters is not exact replication but replication of relevant factors to which I will now turn.

## The broader role of replication in underpinning scientific objectivity

This point can first be illustrated by considering the relation between cookery books and television programmes. Consider Delia Smith's nine step programme for making toast. In line with Collins' discussion, even her careful statement of the process omits a complete list of factors that are not critical but misguided attention to which would stop the process before it began. In general in cookery, knowledge of what is not critical is an important aspect of culinary knowledge. In making pastry, the temperature of the hands and the work top are significant. In preparing a casserole, they are not. Neither, however, are an infinite number of other factors, such as the colour of one's clothes or hair, the phase of the moon etc. Knowing what factors generalise from one process to another and what do not is thus important but is not fully explicit in any cookery manual.

In the face of this challenge, one might retreat to a model of exact copying and turn to the television recordings of Delia Smith actually cooking to preserve an algorithmic model of expertise. Surely, if one mechanically reproduces exactly each stage of the cooking process so recorded one will produce the same degree of success. This assumption is based on a form of supervenience. If culinary results supervene on microphysical properties, then exactly replicating microphysical properties will exactly replicate culinary results.

But consider a practical cookery examination. If a student insists on replicating not only the essential steps in a recipe but also, without discrimination, Smith's choice of oven, sink and kitchen units; the number and make of prepared bowls of ingredients; Smith's style of dress; the view through the window of her kitchen etc this will demonstrate not a fine grained skill but a lack of culinary understanding. Culinary skill involves the ability to replicate all and only relevant factors, not every factor.

Collins develops this point in discussing the role of replication in science more generally [12]. Replication is a central mark of scientific objectivity. In medicine, it lies at the heart of the emphasis on large scale randomised controlled trials and on meta analysis of RCT's. But, as he points out, there are important constraints on what counts as replication.

If there is too close a similarity between one trial and another, then the second does not count as adding evidential weight to the first. If the same scientist takes a second reading from an experimental apparatus a second or two after the first, this does not count as independent confirmation of the first. The test is better if carried out by a different scientist, at a different time, in a different laboratory and so forth. Increasing the differences increases, initially at least, the importance of the replication. But if the 'replication' is carried out by a non-scientist reading tea-leaves then this is a difference too far and again, it does not add evidential weight. Successful evidence-building replication is a compromise between these opposing factors.

Given this, however, evidence-supporting replication is, like culinary skill, a matter of copying relevant factors not exact details. Whilst it is easy to loose sight of this once one ascends to the level of meta-analysis of large scale trials, each individual trial is a kind of experiment and, like the replication of the laser, is an achievement that depends on the controlled deployment of tacit knowledge. Each new trial must be sufficiently like, and sufficiently unlike, the trials that precede it.

The examples drawn from cookery and from applied physics suggest a key role for tacit knowledge. It is not, however, merely the kind of practical know-how involved in dexterous manipulation of the environment. (This is not to play down the role of such know-how in clinical practice.) Rather, even the most cognitive aspects of scientific process turn on tacit knowledge.

## A principled argument for tacit knowledge

The considerations so far have suggested piecemeal arguments for the presence of tacit knowledge in science. But there is stronger argument drawn from Wittgenstein to which I will turn. (Collins also invokes Wittgenstein but to make a distinct claim about the social nature of science.)

Recall the assumption that all genuinely cognitive judgements must be governed by an explicit rule and thus that anything which fails to pass this test is not a case of genuine concept application or valid judgement. This line of argument rests on a mistake, however. It goes wrong in the assumption that it is necessary, or even helpful, to postulate a psychological mechanism to explain an ability to follow a rule. Postulating such a state of mind is an idle wheel because it cannot ground the kind of expectation that either we, or other people, will continue in the same (successful) way.

In an influential central section of his *Philosophical Investigations*, Ludwig Wittgenstein discusses rule following in detail. Taking the case of continuing a mathematical series, he considers how it is possible to teach or to learn how to go on correctly, given the infinite nature of the task. Consider the problem of ascribing knowledge of the correct continuation to another person. Given the infinite number of variations in principle available at some higher number (eg responding to the instruction "add 2" by continuing 996, 998, 1000, but then 1004, 1008 etc) it may seem epistemically risky to ascribe shared understanding of the correct continuation on the basis of finite past practice. Hence the temptation to ascribe a psychological mechanism to explain the ability and thus narrow down the range of future moves.

As Wittgenstein shows, however, if inferring from finite past to future practice were not reliable in itself, postulating the intervening mechanism would not help either. Past practice, once described as mere *inductive *evidence for a mechanism, could be evidence for any number of diverging mechanisms. Any finite number of examples might be interpreted to be in accord with an infinite number of possible continuations. It is easiest to think of this in the case of a third person ascription to another. But there are similar problems in the first person case as well in ensuring that one embodies just the right mechanism oneself.

In both cases, the felt need for an *interpretation *leads to a vicious regress. In the former case, other people's past practice has to be interpreted as following a particular rule. In the latter, one's own mental states, mental signs or processes have to be interpreted as determining a rule. Hence Wittgenstein concludes:

This was our paradox: no course of action could be determined by a rule, because every course of action can be made out to accord with the rule. The answer was: if everything can be made out to accord with the rule, then it can also be made out to conflict with it. And so there would be neither accord nor conflict here. It can be seen that there is a misunderstanding here from the mere fact that in the course of our argument we give one interpretation after another; as if each one contented us at least for a moment, until we thought of yet another standing behind it. What this shews is that there is a way of grasping a rule which is *not *an *interpretation*, but which is exhibited in what we call "obeying the rule" and "going against it" in actual cases. [13]

A key move to avoid the problem turns on rejecting the view of theory neutral evidence for another person's grasp of a rule. Instead, as long as one adopts the same perspective then one can see in the practice the rule that is exemplified. That we are able to adopt the same perspective depends on the fact that we share the same 'whirl of organism', in Stanley Cavell's memorable phrase [14].

Thus even judgements that can be codified such as continuing a mathematical series rest on tacit knowledge. What then should be said about hard cases: judgements that are not codified? Under the grip of the prejudice that Wittgenstein exposes, one might assume that either there is a piece of knowledge that could in principle be articulated or it is not a case of conceptual judgement. But since the proper understanding of even codifiable judgement shows that what is primitive is a notion of going on in the same way which cannot be explained as the result of a mechanism, the contrast between the two cases is diminished. One way of putting this point is to suggest an equivalence between a hard case for uncodifiable judgement and what could be said if someone were to question how to apply the series add 2 at the point at which, in Wittgenstein's phrase, justifications have come to an end. ('Giving grounds, however, justifying the evidence, comes to an end; – but the end is not certain propositions' striking us immediately as true, i.e. it is not a kind of *seeing *on our part; it is our acting which lies at the bottom of the language game' [15])

Drawing on Wittgenstein's discussion, McDowell suggests that the model of uncodified conceptual judgement is this:

We are inclined to be impressed by the sparseness of the teaching that leaves someone capable of autonomously going on in the same way. All that happens is that the pupil is told, or shown, what to do in a few instances, with some surrounding talk about why that is the thing to do; the surrounding talk, *ex hypothesi*... falls short of including actual enunciation of a universal principle, mechanical application of which would constitute correct behaviour in the practice in question. Yet pupils do acquire a capacity to go on, without further advice, to novel instances. Impressed by the sparseness of the teaching, we find this remarkable. [16]

If there is no universal principle then it can seem miraculous that finite teaching enables students to go on in the same way. But this is a misleading contrast because if there is a universal principle, it would be equally miraculous how they grasp the *right *principle from finite teaching.

The moral of Wittgenstein's discussion of rules in this case is this. Given that discussion, even in the case of judgements for which a universal principle can be written down, the ability to apply the principle depends on our basic responses to it. Justifications for particular responses to a rule come to an end. Fortunately because humans share the same basic reactions, responses, routes of salience and so on, they typically come to an end in agreement. But this makes the idea that there are uncodifiable judgements innocuous. Both codified and uncodified judgements are, ultimately, in the same boat. Both depend on a tacit element.

Wittgenstein's discussion thus provides a powerful argument about the role of tacit knowledge in judgement. At the heart of explicit codified knowledge is judgement in accordance with rules. But whatever can made explicit in specifying what does and what does not accord with a rule must itself inevitably rest on something implicit and tacit. (The later Wittgenstein is not the only source for principled arguments for the role of tacit knowledge. Other sources include the early Heidegger and more recent phenomenology, which is particularly resonant for issues in mental health care, as well as arguments for moral particularism drawing on Aristotle.)

## Tacit knowledge and explicit guidelines in medical research and practice

I have argued that research drawn from the history of science, the sociology of science and pure philosophy shows that there is an element of tacit knowledge, of practical know-how, underpinning the most explicitly codified elements of EBM: research evidence driven by scientific method. Arguments drawn from Collins' analysis of empirical replication and the argument from Wittgenstein's consideration of rule governed judgement show that such tacit knowledge is in principle ineliminable.

This is not to say, however, that codifications can play no useful practical roles. Consider, for example, the guidelines set out by Pocock on clinical trials in his *Clinical Trials: A Practical Approach*. He stipulates that trials should be:

**comparative**. The experiences of patients on the treatment under trial are compared with a control group: the experiences of patients on other treatments (possibly including no treatment).

**randomized**. This is supposed to prevent conclusions being drawn about the effects of drug treatments, for example, which are really the effects of some other uncontrolled for factor present in the sample.

**double blind**, wherever possible. Neither the patients nor the clinicians testing results should know whether they belong to the test group or control group.

Aside from these general features, Pocock claims that clinical trials should proceed through a pre-determined series of steps 'if the principles of scientific method are to be followed' [17]. Those steps are:

1. Define the purpose of the trial: state specific hypotheses.

2. Design the trial: a written protocol.

3. Conduct the trial: a good organisation.

4. Analyse the data: descriptive statistics, tests of hypotheses.

5. Draw conclusions: publish results

These are useful instructions and the emphasis on randomized comparative and double blind trials fits with EBM.

The account of scientific method, though clearly only describing a small element of scientific practice, might also be a useful summary of the current phase of medical 'normal science'. But it is a useful summary only for someone able to follow it. Point 2 may be a useful corrective for anyone unused to having to 'show their workings'. Point 3: 'conduct the trial', gnomically summarises in three words Bob Harrison's lengthy struggles as set out by Collins. Point 4 omits an account of *how *to analyse data which might take more than one university course. Point 5 omits the further efforts involved in genuinely publishing results described by Collins: the personal contact and instruction and shared experience of working experimental setups etc.

Two general points are worth noting here. First, a natural response would be to say that this is just a brisk list of headings and that each step can be further described (which is precisely the aim of books such as Pocock's). But the arguments in the rest of this paper show that whatever is made explicit in further explanations cannot in itself be sufficient to determine correct practice. That also requires a shared background of tacit knowledge. Whatever is made explicit, something is always left implicit.

Second, however, providing one does have a sufficient background of tacit skill, even a recipe as brisk as the above list might well be a useful reminder of the steps to be gone through and their order. The fact that tacit knowledge is a necessary element of scientific judgement does not undermine a practical use for some codifications. But it does suggest a principled limit to the ambition to codify all that is involved in having good judgement.

The same general lessons apply, for example, to attempts to codify diagnosis in general practice. Diagnosis has been codified as a 'hypothetico-deductive' process [18]. In clinical handbooks, this process is sometimes represented in a flow diagram which starts with the inputs to the process (clinical background knowledge, and presenting signs and symptoms) and progresses via a list of hypotheses that are tested against further information gathering resulting in a plan for medical management. Such flow charts can be useful prompts or reminders but this is not because they fully capture diagnostic expertise in temselves. The boxes describing the inputs of background medical knowledge and patient signs and symptoms have to be interpreted in the light of clinical skill and experience. How much is enough background medical knowledge? How many degrees should one take? How long should one give a patient to describe symptoms? How many possible hypotheses should be actively considered at each stage? Practical teaching helps fill in the answers to these questions whether by prompting an explicit answer (eg suggesting a range of 3–5 hypotheses) or implicitly guiding practice (eg the skilled clinician can judge how much time different patients in context), or by ruling out an inquiry that could be raised (eg simply silencing a potential worry about the number of medical degrees required).

In a context of such background tacit knowledge, filling in the gaps in the codification becomes second nature. But whilst this may disguise the role of tacit knowledge it does not eliminate its need. Providing they are interpreted intelligently, diagrammatic codifications can be helpful guides to practice. What they cannot do, however, is capture good clinical judgement independently of the tacit background. In themselves they remain merely partial and schematic codifications of practice.

## Conclusion

The model of judgement as an uncodified exercise of practical rationality provides a way to unify Sackett et al's tripartite broader definition of EBM as the integration of best research evidence with clinical expertise and patient values. Whilst it is tempting to contrast the explicit knowledge-base of research evidence with the implicit skills of clinical expertise – downplayed by most accounts of EBM – and the necessary judgement involved in balancing opposing values in Values-Based Practice (when properly understood), all depend on an ability to exercise judgement. This is so even though it is often hidden by skill and practice.

In fairness, the best accounts of the practice of EBM do not present even the research evidence element as algorithmic (a good short account is [19]). The problem, however, is that this tends to be forgotten in the face of the prejudice about rationality I outlined at the start of this paper. In practice, in other words, the need for judgement is downplayed in the face of rising numbers of explicit guidelines and codifications setting out good practice.

In this respect, EBM resembles the version of principlism in medical ethics set out by Beauchamp and Childress. In *Principles of Biomedical Ethics*, Beauchamp and Childress stress the need for judgement in the application of the principles they set out [20]. In practice, however, despite the intentions of its authors, the Four Principles Approach to medical ethics can quickly seem to promise an algorithmic model of ethical competence [3]. In part this is because insufficient attention is paid to how ethical judgements might not be fully codified in principles and yet still be an exercise of objectivity and rationality.

In a similar way, despite the emphasis on the role of judgement in EBM by some authors, it can drop from view when presented with the sheer weight of trial numbers summarised in a Cochrane review. But by looking back at the hidden details of scientific process behind that data and by reflecting on what is involved in correctly following explicit rules in applying it in context, the fundamental role of tacit knowledge can be brought to view again. There is an ineliminable role for uncodified and tacit knowledge, or judgement, behind even explicit research evidence. Tacit knowledge serves to unify all three of Sackett's tripartite articulation of EBM. In a slogan, at the heart of evidence based medicine is good judgement.

One further connection is worth making. The main claim of this paper, that even explicit research-driven evidence-based medicine rests on a background of implicit or tacit knowledge, has echoes with another general change in theoretical orientation in medical practice. Emphasising the role of tacit knowledge plays up the idea that those who make medical judgements are not abstract rational points of view but embodied agents who share a 'whirl of organism'. It is this shared practical orientation that underpins abstract conceptual judgement and reliability between different clinicians.

This parallels another influential recent idea: the rise of the importance of user centred care. User centred care contrasts with an over concentration on symptoms considered in isolation. The idea of user centred care also has a philosophical analogue which is particularly germane to *mental *health care which might be expressed in the slogan: the smallest unit of meaning is the life of the whole person. (This view contrasts with a focus instead on trying to explain the meaning of either individual brain states or mental representations through causal connections. That reductionist programme faces severe conceptual difficulties, however [21].)

The underlying metaphysics of user centred care is the patient as a whole person, the meaning of whose life is a structured – though in mental illness perhaps fractured – whole. The metaphysics of tacit knowledge is one of clinicians as embodied whole persons exercising judgement in the face both of complex data and guided by only partial codifications. These two views fit together with a much more humane account of the relation of 'subject' and 'object' in clinical judgement: a relation that will be better understood through a contribution from philosophy and the humanities as well as the hard sciences.
